# Pneumococcal nasopharyngeal carriage among Bhutanese children hospitalized with clinical pneumonia: serotypes and viral co-infection

**DOI:** 10.1186/s12879-020-05674-4

**Published:** 2020-12-09

**Authors:** Sophie Jullien, Ragunath Sharma, Mimi Lhamu Mynak, Desiree Henares, Carmen Muñoz-Almagro, Quique Bassat

**Affiliations:** 1 Barcelona Institute for Global Health (ISGlobal), Hospital Clinic, Universitat de Barcelona, Barcelona, Spain; 2 Jigme Dorji Wangchuck National Referral Hospital, Thimphu, Bhutan; 3 Instituto de Recerca Pediatrica, Hospital Sant Joan de Deu (University of Barcelona), Barcelona, Spain; 4 CIBER of Epidemiology and Public Health CIBERESP, Madrid, Spain; 5 Department of Medicine, Universitat Internacional of Catalunya, Barcelona, Spain; 6 Institució Catalana de Recerca i Estudis Avançats, Barcelona, Spain; 7 Pediatric Infectious Diseases Unit, Pediatrics Department, Hospital Sant Joan de Déu (University of Barcelona), Barcelona, Spain; 8 Centro de Investigação em Saúde de Manhiça (CISM), Maputo, Mozambique

**Keywords:** *Streptococcus pneumoniae*, Pneumonia, Colonization, Bhutan, Child preschool

## Abstract

**Background:**

Pneumococcal nasopharyngeal colonization (PNC) generally precedes pneumococcal disease. The purpose of this study was to determine the prevalence of PNC and to identify the pneumococcal serotypes circulating among Bhutanese children under five years of age admitted with clinical pneumonia, before the introduction of pneumococcal conjugate vaccine (PCV13) in the country. We also aimed to contribute to the understanding of the interplay between PNC and viral co-infection among this population.

**Methods:**

This was a prospective study conducted at the Jigme Dorji Wangchuck National Referral Hospital in Bhutan over 12 consecutive months. Children aged 2 to 59 months admitted with WHO-defined clinical pneumonia were eligible for recruitment. We collected blood for bacterial culture and molecular identification of *S. pneumoniae*, and nasopharyngeal washing for screening of respiratory viruses, and for the detection and capsular typing of *S. pneumoniae* by real-time polymerase chain reaction (RT-PCR).

**Results:**

Overall, 189 children were recruited, and PNC was tested in 121 of them (64.0%). PNC was found in 76/121 children (62.8%) and *S. pneumoniae* was identified in blood (both by culture and RT-PCR) in a single child. Respiratory viruses were detected in a similar proportion among children with (62/70; 88.6%) and without PNC (36/40; 90.0%; *p* = 1.000), but rhinovirus detection was less common among children with PNC (20/70; 28.6% versus 19/40; 47.5%; *p* = 0.046). Capsular typing identified 30 different serotypes. Thirty-nine children (51.3%) were colonised with two to five different serotypes. A third of the children presented with serotypes considered highly invasive. Over half of the children (44/76; 57.9%) were carrying at least one serotype included in PCV13.

**Conclusions:**

This study provides baseline information on the status of PNC among Bhutanese children admitted with clinical pneumonia prior to the introduction of PCV13, which is valuable to monitor its potential impact. PCV13 could theoretically have averted up to 58% of the pneumococcal infections among the children in this study, suggesting a future role for the vaccine to significantly reduce the burden associated with *S. pneumoniae* in Bhutan.

## Background

*Streptococcus pneumoniae* is a common cause of invasive bacterial disease (IBD), including pneumonia, meningitis and sepsis in children under five years of age. Although the prevalence of pneumococcal associated IBD and deaths has declined in the last two decades, mainly as a result of the parsimonious global introduction of pneumococcal conjugate vaccines (PCV), this pathogen still causes a significant burden. In 2015, *S. pneumoniae* was estimated to cause 8.9 million cases of clinical pneumonia in children aged 1 to 59 months, and 294,000 deaths among HIV-uninfected children aged 1 to 59 months. The majority of these deaths (81%) were attributed to pneumonia [1]. The vast majority of the pneumococcal burden is now concentrated in low- and middle-income countries (LMIC). In 2015, approximately 50% of all pneumococcal deaths were registered in four countries: India, Nigeria, the Democratic Republic of the Congo, and Pakistan [1].

Pneumococcal nasopharyngeal colonization (PNC) is generally considered a prerequisite for pneumococcal disease and is a source of spread between people [2, 3]. However, *S. pneumoniae* is part of the commensal nasopharyngeal flora and in most instances, PNC does not lead to disease [3]. While the association between PNC and development of acute otitis media is well recognised, its relationship with pneumonia is less strongly established [4, 5]. Other bacteria and viruses are common colonizers of the nasopharynx. The interplay between respiratory viruses and *S. pneumoniae* on the progression to the disease is still poorly understood [6, 7].

By December 2019, 145 countries had introduced PCV into their national immunization programme and 15 additional countries were planning to do so [8]. Currently, there are two WHO prequalified vaccines that are commonly used: PCV13 (Prevenar 13®, Pfizer) and PCV10 (Synflorix®, GlaxoSmithKline) that include 13 and 10 serotypes respectively [9, 10]. A third vaccine that also includes 10 serotypes (Pneumosil®, Serum Institute of India) was recently prequalified by WHO in December 2019 [9]. The introduction of PCV has substantially reduced both the burden of pneumococcal invasive disease and the rates of PNC by serotypes included in the vaccine [1, 11–14]. The emergence of serotypes not included in the vaccines has been well documented in high-income countries but little is known in LMIC [15–17], and surveillance data at a national level are important to identify serotype replacement and to assess most prevalent serotypes still circulating in the population.

Bhutan is a small country landlocked in the Himalayas, with an estimated population of 779,666 in 2017 [18, 19]. It is currently classified as a lower-middle income country [20]. The Constitution guarantees free essential health services for Bhutanese citizens, based on a primary health care approach [21]. Similar to other LMIC, pneumonia remains a major public health challenge in Bhutan, whereby the number of outpatient visits and hospitalizations attributed to pneumonia constitutes a considerable burden to the health system [22]. The conjugate *Haemophilus influenza type b* (Hib) vaccine has been routinely administered since 2011, and PCV13 was introduced in the immunization programme in January 2019 [23, 24]. Despite the burden that pneumonia represents for the country, there are scarce national data on the epidemiology and aetiology of childhood pneumonia, leading to challenges while implementing effective national preventive strategies [25]. Furthermore, while information on the pneumococcal serotypes circulating before the introduction of PCV13 is essential to monitor the impact of the vaccine, there are no data on circulating pneumococcal serotypes among the Bhutanese population [25].

We conducted this prospective study to determine the prevalence of pneumococcal carriage and to identify the pneumococcal serotypes circulating among Bhutanese children under five years of age admitted with WHO-defined pneumonia, before the introduction of PCV in the country. We also aimed to contribute to the understanding of the interplay between PNC and viral co-infections among this population.

## Methods

### Study design and patient enrolment

This prospective Respiratory Infection in Bhutanese Children (RIBhuC) study was conducted over 12 consecutive months at the Jigme Dorji Wangchuck National Referral Hospital (JDWNRH) in Thimphu, Bhutan. The RIBhuC study aimed to describe the epidemiology, aetiology and clinico-radiological presentation of WHO-defined pneumonia among admitted children under five years of age. The recruitment process and data collection have been described elsewhere [26]. In brief, we recruited all children aged 2 to 59 months who were admitted at JDWNRH with a diagnosis of pneumonia (including severe pneumonia) according to the WHO definitions [27]. Pneumonia was defined as history of cough or reported breathing difficulty, together with increased respiratory rate (respiratory rate ≥ 50 breaths per minute in children aged 2 to 11 months; or respiratory rate ≥ 40 breaths per minute in children aged 12 to 59 months) or chest indrawing. Severe pneumonia was defined as history of cough or reported breathing difficulty, and at least one of the following: oxygen saturation < 90% or central cyanosis, severe respiratory distress (e.g. grunting, very severe chest indrawing), or general danger sign (inability to breastfeed or drink, lethargy or reduced level of consciousness, convulsions). Children admitted in the previous seven days were not recruited to the study in order to exclude hospital-acquired infections. Children whose principal reason for admission was a non-respiratory illness or a condition that was not caused by respiratory illness, and those with evidence of a foreign body in the respiratory tract were also excluded.

### Data collection

For all eligible patients whose parents consented to participate in the study, we performed a meticulous physical examination and collected biological samples at time of admission (or as soon as possible after admission) and before initiation of antibiotics. This included blood samples for bacterial culture, full blood cell count, and biochemistry; and nasopharyngeal washing (NPW) for the identification of *S. pneumoniae*, respiratory viruses and atypical bacteria. We collected demographic and clinical data from the medical records and by interviewing the parents. A chest radiography (CXR) was indicated for each child on admission.

### Specimen collection and laboratory testing

#### Nasopharyngeal sample collection and storage

Respiratory secretions were collected through NPW, using 1 to 3 mL of 0.9% saline solution with a commercial mucus extractor kit, and sent to the local microbiology lab within 30 min, according to the corresponding standard of procedure developed in our protocol [28]. Specimens were homogenized, aliquoted, frozen at − 80 °C, and shipped to Hospital Sant Joan de Déu in Barcelona, Spain, for centralised molecular analyses.

#### Pneumococcal detection and capsular typing from respiratory secretions

We performed a duplex real-time polymerase chain reaction (RT-PCR) targeting the *lytA* gene of *S. pneumoniae* and the internal control targeting RNaseP of human cells for DNA amplification, using the Applied Biosystems 7500 RT-PCR System (Applied Biosystems, CA, US) [29]. We performed capsular typing of *S. pneumoniae* in all *lytA* positive samples with a fragment analysis multiplex PCR for distinguishing 40 serotypes [30]. We considered the serotypes 1, 3, 4, 5, 7F, 14, 18C and 19A as highly invasive according to findings from other studies [31–36], and refer to the remaining serotypes as ‘less-highly invasive’.

#### Detection of respiratory viruses and atypical bacteria from respiratory secretions

For identification of respiratory viruses and atypical bacteria, we used the multiplex RT-PCR QIAStat respiratory panel, Qiagen, which includes 17 viral targets (adenovirus, bocavirus, coronavirus 229E/HKU1/NL63/OC43, human metapneumovirus, influenza virus A/B [A subtypes H1N1pdm09, H1, H3], parainfluenza viruses 1/2/3/4, respiratory syncytial virus, rhinovirus) and four bacteria (*Bordetella pertussis*, *Chlamydophila pneumophila*, *Legionella pneumophila*, and *Mycoplasma pneumoniae*) [37, 38].

#### Blood collection and testing

We collected blood for haematology, biochemistry and bacterial culture. Blood samples were processed at JDWNRH following standardized procedures. Blood was cultured using an automated blood culture system (BacT/ALERT®), and bacterial isolates were identified by colony morphology, growth requirements, and basic biochemical tests. Two drops of blood were collected on filter paper and shipped to Spain for further screening of *S. pneumoniae* (LytA gene) by RT-PCR.

### Chest radiography interpretation

The methods we used for the interpretation of CXR are described in detail elsewhere [26]. In brief, we followed the WHO protocol used in clinical trials of PCV [39]. Two paediatricians independently assessed each CXR, a third reader read the discordant results, and a paediatric radiologist interpreted again all CXR for additional reliability. CXRs were classified as either radiologically confirmed endpoint pneumonia (defined as consolidation, pleural effusion or both on any hemithorax), other infiltrates, or normal.

### Data management and statistical analysis

The lead investigator entered data into a computerized password-protected database (ODK®) with study identification number. We limited errors in data entry by pre-defining ranges for every value. We used Stata 16.0 for data analysis [40]. We examined the association between pneumococcal carriage and a set of clinical signs and potential risk factors by using Chi-square or Fisher exact test (for categorical variables) and Wilcoxon rank-sum test (for continuous variables). A *p*-value less than 0.05 was considered statistically significant.

## Results

**Fig. 1:**
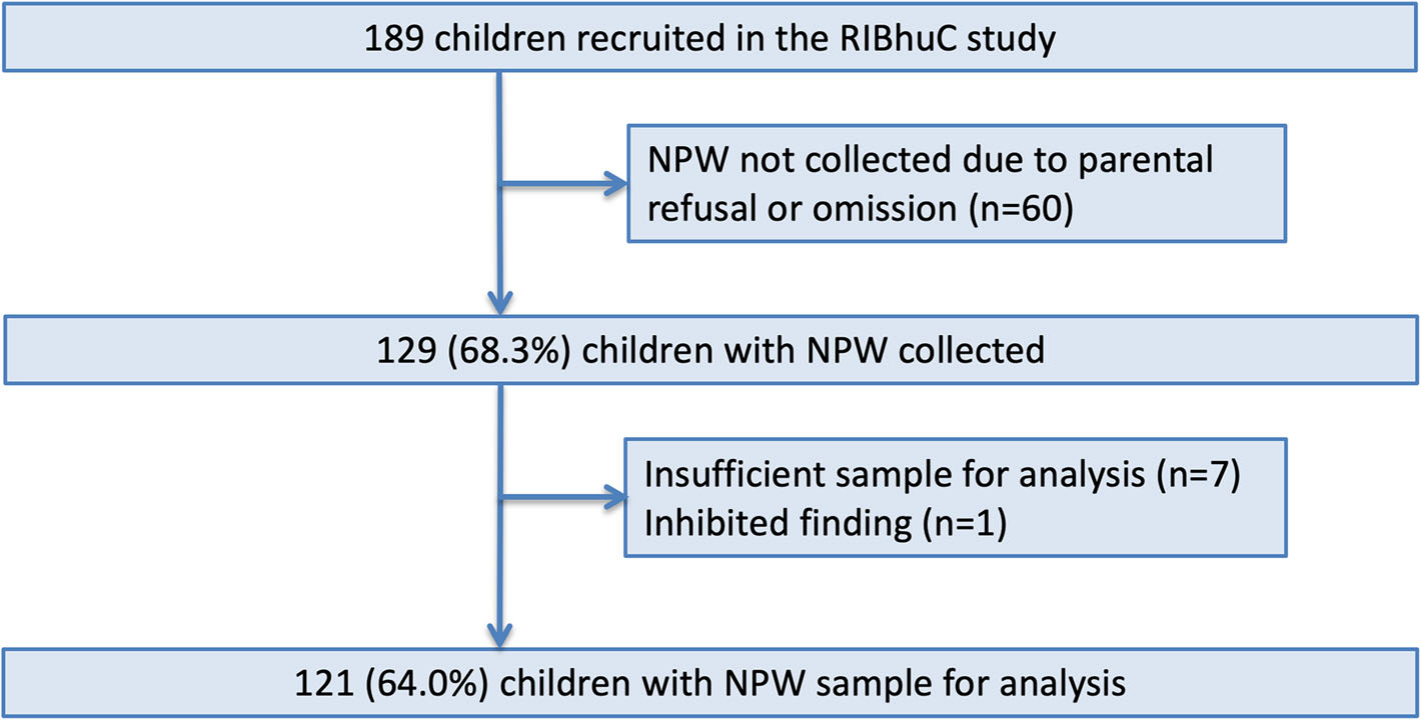
Study profile. Abbreviations: NPW: nasopharyngeal washing; RIBhuC: Respiratory Infections in Bhutanese Children

Among the 189 children recruited in the RIBhuC study between 1st July 2017 and 30th June 2018 [26], NPW was collected in 129 children (68.3%). After microbiological screening for virus, there was insufficient sample for pneumococcal testing in seven children, and one sample showed an inhibited finding, leaving 121/189 cases (64.0%) for analysis (Fig. 1).

### Pneumococcal nasopharyngeal carriers’ prevalence and characteristics

Pneumococcal carriage was found in 76/121 children (62.8%). We reported the baseline characteristics of children by status of nasopharyngeal colonization of *S. pneumoniae* in Table 1. Among colonized children, around half of them were infants (54.0%), and this proportion of infants was similar in the non-colonized (53.3%) group. Among colonized children and compared to non-colonized ones, there was a significant higher proportion of females (38/76, 50.0%; versus 13/45, 28.9%; *p* = 0.023).

**Table 1:** Baseline characteristics of children with and without pneumococcal nasopharyngeal colonization

	***S. pneumoniae*** colonization ***n*** = 76 (62.8%)	No ***S. pneumoniae*** colonization ***n*** = 45 (37.2%)	***p***-value^**a**^
**Demographic characteristics**			
Gender, female	38/76 (50.0%)	13/45 (28.9%)	0.023
Age in months (median, IQR)	10.8 (7.6–23.4)	9.7 (4.0–23.0)	0.169
Age groups			0.948
Infants (< 12 months)	41/76 (54.0%)	24/45 (53.3%)	
≥ 12 months	35/76 (46.0%)	21/45 (46.7%)	
HIV infection	0/76 (0%)	0/45 (0%)	NA
Vaccination status			0.157
Fully vaccinated according to age	63/75 (84.0%)	33/45 (73.3%)	
Partially vaccinated according to age	12/75 (16.0%)	12/45 (26.7%)	
Not vaccinated	0/75 (0%)	0/45 (0%)	
At least one other child under 5 years of age in the household	32/76 (42.1%)	14/45 (31.1%)	0.229
6 people or more living in the household	30/76 (39.5%)	16/45 (36.4%)	0.736
Education			0.157
Both parents are illiterate	10/75 (13.3%)	8/44 (18.2%)	
Only one parent has primary education	17/75 (22.7%)	4/44 (9.1%)	
Both parents have primary education	28/75 (37.3%)	23/44 (52.3%)	
At least one parent has university education	20/75 (26.7%)	9/44 (20.4%)	
Employment			1.000
Both parents are unemployed	1/71 (1.4%)	1/43 (2.3%)	
Only one parent is employed	46/71 (64.8%)	27/43 (62.8%)	
Both parents are employed	24/71 (33.8%)	15/43 (34.9%)	
Season			0.677
Summer	27/76 (35.5%)	15/45 (33.3%)	
Fall	30/76 (39.5%)	16/45 (35.6%)	
Winter	5/76 (6.6%)	6/45 (13.3%)	
Spring	14/76 (18.4%)	8/45 (17.8%)	
**History and severity of the current episode**			
Antibiotics started prior to admission	10/75 (13.3%)	14/45 (31.1%)	0.018
Antibiotics prior to NPW collection			< 0.001
No	52/76 (68.4%)	13/45 (28.9%)	
Yes, for less than 24 h	13/76 (17.1%)	14/45 (31.1%)	
Yes, for more than 24 h	11/76 (14.5%)	18/45 (40.0%)	
Days of fever prior admission (median, IQR)	3 (1–5)	2 (0–4)	0.363
WHO severe pneumonia on admission	55/76 (72.4%)	38/45 (84.4%)	0.256
Severe pneumonia during admission	60/76 (79.0%)	39/45 (86.7%)	0.287
**Radiological findings**			
CXR findings			0.051
Pneumonia endpoint	20/67 (29.9%)	10/38 (26.3%)	
Other infiltrates	7/67 (10.4%)	11/38 (29.0%)	
Normal	40/67 (59.7%)	17/38 (44.7%)	
**Laboratory findings**			
CRP > 4 mg/dL	10/73 (13.7%)	8/42 (19.1%)	0.447
ESR ≥ 50 mm	13/69 (18.8%)	4/39 (10.3%)	0.239
Leucocytosis	34/76 (44.7%)	15/44 (34.1%)	0.253
Neutrophilia	28/76 (36.8%)	9/44 (20.5%)	0.061
**Evolution and outcome**			
Oxygen therapy during hospitalization	52/76 (68.4%)	34/45 (75.6%)	0.403
Duration of hospitalization			0.285
< 24 h	3/76 (3.9%)	2/45 (4.4%)	
≥ 24 to < 72 h	31/76 (40.8%)	12/45 (26.7%)	
≥ 72 h to < 7 days	29/76 (38.2%)	25/45 (55.6%)	
≥ 7 days	13/76 (17.1%)	6/45 (13.3%)	
Fatal outcome	1/76 (1.3%)	1/45 (2.2%)	1.000
Poor prognosis, simple definition (admission to PICU and/or fatal outcome)	10/76 (13.2%)	7/45 (15.6%)	0.714
Extended poor prognosis definition (admission to PICU, admission to HDU, fatal outcome, and/or hospitalization ≥7 days)	23/76 (30.3%)	15/45 (33.3%)	0.725

***Abbreviations*:** *CRP* C-reactive protein, *CXR* chest radiography, *ESR* erythrocyte sedimentation rate, *HDU* high dependency unit, *IQR* interquartile range, *NA* not applicable, *PICU* paediatric intensive care unit

^a^We examined the association between pneumococcal carriage status and the selected variables using Chi-square or Fisher exact test (for categorical variables) or Wilcoxon rank-sum test (for continuous variables non-normally distributed)

There was no significant difference in the proportion of children with at least another child under five years of age in the same household between colonized and non-colonized children (42.1% versus 31.1%; *p* = 0.229). Colonized children were less likely to have received antibiotics prior to admission (13.3% versus 31.1%; *p* = 0.018) and prior to NPW specimen collection (31.6% versus 71.1%; *p* = < 0.001).

On CXR, there was a trend for children with PNC to present less infiltrates than those without colonization (10.4% versus 29.0%), but there was no differences in the proportion of children with endpoint pneumonia (29.9% versus 26.3%; *p* = 0.051).

There were no significant differences between colonizers and non-colonizers in regard to laboratory findings (such as C-reactive protein or erythrocyte sedimentation rate), outcome, and prognosis.

### Microbiological findings by pneumococcal nasopharyngeal colonization

#### Bacterial findings

Overall, six children had bacteria isolated by blood culture (five with PNC and one without PNC), of which only one was *S. pneumoniae* (Table 2).

**Table 2:** Bacterial and viral findings by pneumococcal nasopharyngeal colonization

	No ***S. pneumoniae*** colonization ***n*** = 45 (37.2%)	***S. pneumoniae*** colonization ***n*** = 76 (62.8%)	***p***-value^**a**^
**Bacterial findings**			
Positive bacterial blood culture (of any cause)	1/39 (2.6%)	5/62 (8.1%)^b^	0.401
Positive *S. pneumoniae* (blood culture)	0/39 (0%)	1/62 (1.6%)	1.000
Positive *S. pneumoniae* (dry blood spot, RT-PCR)	0/38 (0%)	1/64 (1.6%)	1.000
Positive for atypical bacteria in NPW			
Bordetella pertussis	1/40 (2.5%)	2/70 (2.9%)	1.000
Chlamydophila pneumophila	0/40 (0%)	0/70 (0%)	NA
*Legionella pneumophila*	0/40 (0%)	0/70 (0%)	NA
Mycoplasma pneumoniae	0/40 (0%)	1/70 (1.4%)	1.000
**Viral findings**			
At least one virus identified in NPW	36/40 (90.0%)	62/70 (88.6%)	1.000
Multiple (≥2) viruses identified in NPW	13/36 (36.1%)	20/62 (32.3%)	0.697
Positive for respiratory syncytial virus	13/40 (32.5%)	35/70 (50.0%)	0.075
Positive for rhinovirus	19/40 (47.5%)	20/70 (28.6%)	0.046
Positive for influenza virus	8/40 (20.0%)	8/70 (11.4%)	0.220
Positive for parainfluenza virus	5/40 (12.5%)	13/70 (18.6%)	0.408
Positive for adenovirus	3/40 (7.5%)	5/70 (7.1%)	1.000
Positive for bocavirus	4/40 (10.0%)	2/70 (2.9%)	0.188
Positive for human metapneumovirus	2/40 (5.0%)	2/70 (2.9%)	0.621
Positive for coronavirus	1/40 (2.5%)	1/70 (1.4%)	1.000

***Abbreviations*:** *NPW* nasopharyngeal washing, *RT-PCR* real-time polymerase chain reaction

^a^We compared the proportions of the selected variables between pneumococcal colonization and non-colonization using Chi-square or Fisher exact test (for categorical variables) or Wilcoxon rank-sum test (for continuous variables non-normally distributed)

^b^One culture positive to *S. pneumoniae*

*S. pneumoniae* was isolated by blood culture in a single child, and subsequently confirmed by molecular methods (RT-PCR in dried blood spot). That same child was found to be a pneumococcal carrier in the nasopharynx.

#### Association of PNC with viral co-infection

Respiratory viruses were detected in a similar proportion among children with and without PNC (62/70; 88.6% versus 36/40; 90.0%; *p* = 1.000) (Table 2). However, rhinovirus detection was more common among children without PNC (47.5% in non-colonised children versus 28.6%; *p* = 0.046), whereas respiratory syncytial virus (RSV) was more common in the colonized group, although this did not reach statistical significance (50.0% in colonized children versus 32.5%; *p* = 0.075). No further significant differences were found regarding the detection of other viruses.

### Distribution of pneumococcal serotypes

**Fig. 2:**
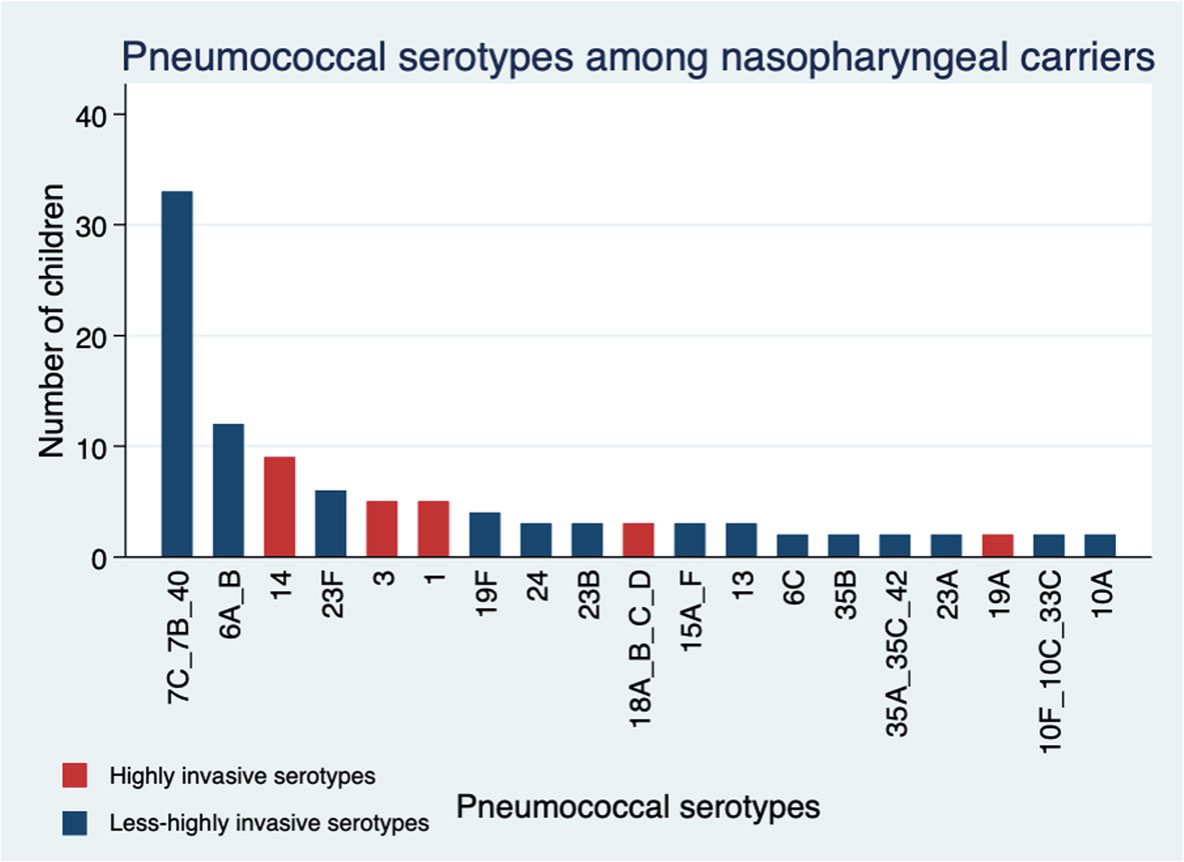
Bar graphs of pneumococcal serotypes among nasopharyngeal carriers. Other serotypes were identified in one child each: high invasive (4, 5, 7F/A, and 15B/C) and non-high invasive (9 V/A, 16F, 20, 22A/F, 34, 35F/47F, and 39). Among the 12 children presenting with the serotype 6A/B, 2 cases were serotype 6A, 2 cases were serotype 6B, and it was not possible to differentiate between 6A and 6B in the remaining 8 cases

Thirty different serotypes (or groups of serotypes when it was not possible to differentiate them) were identified among the 76 children with PNC (Fig. 2). Over half of the children (39/76; 51.3%) were colonized with at least two and up to five different serotypes. The less-highly invasive serotypes 7B/C or 40 (the laboratory technique being unable to differentiate between these three serotypes) were the most common identified, being detected in 33/76 children (43.4%). The following most common less-highly invasive serotypes were 6A/B (12/76; 15.8%), 14 (9/76; 11.8%), and 23F (6/76; 7.9%). Other serotypes not included in the multiplex PCR technique used for this study were found in 10/76 children (13.2%).

**Fig. 3:**
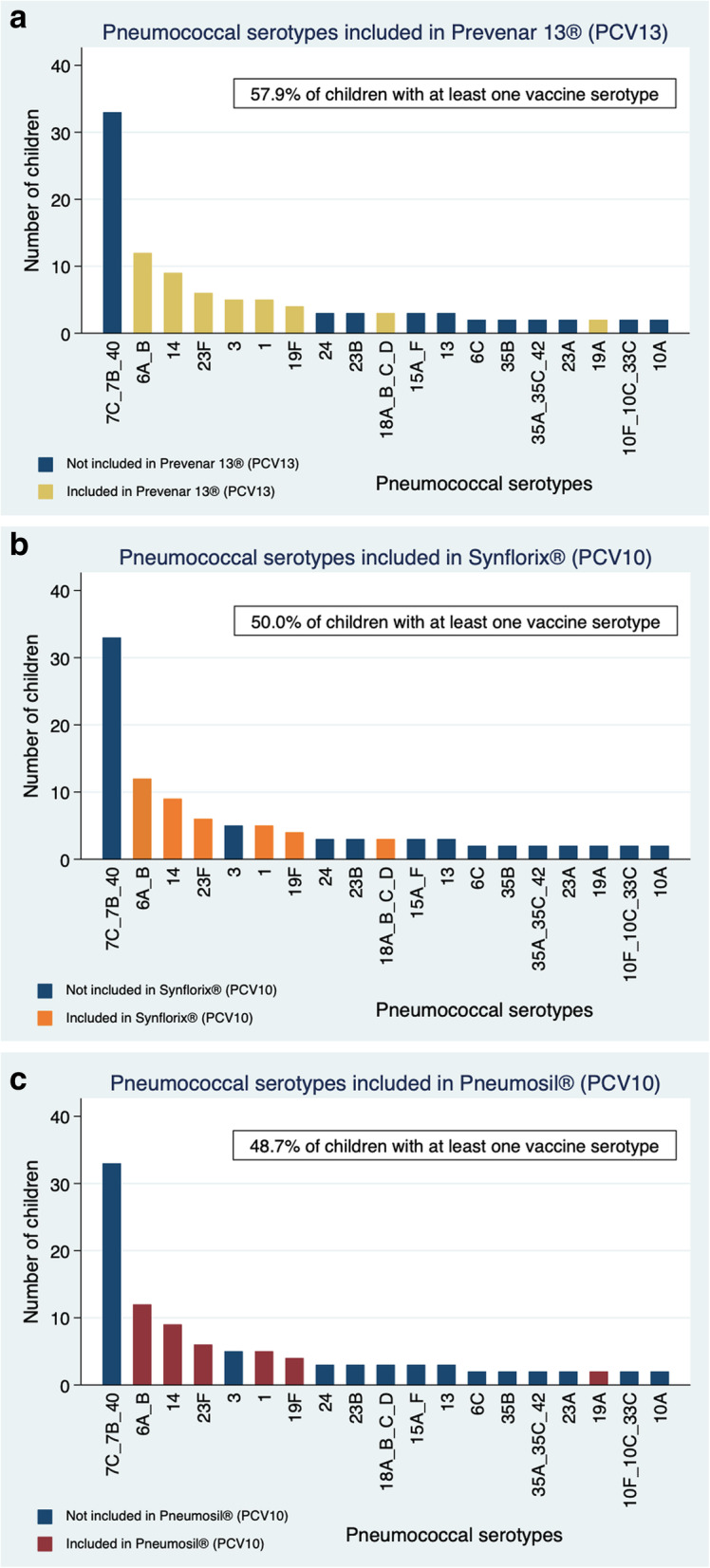
Bar graphs of pneumococcal serotypes included in WHO pre-qualified PCV. **a**. Pneumococcal serotypes included in Prevenar 13® (PCV13). **b**. Pneumococcal serotypes included in Synflorix® (PCV10). **c**. Pneumococcal serotypes included in Pneumosil® (PCV10). We identified 3 children with serotype 18A/B/C/D, the laboratory technique not being able to differentiate among 18A, 18B, 18C or 18D. However, Prevenar 13® and Synflorix® only include the serotype 18C. While Prevenar 13® include both serotypes 6A and 6B, Synflorix® only include the serotype 6B. We identified 2 children with serotype 6A; 2 children with serotype 6B, and it was not possible to differentiate between 6A and 6B in the remaining 8 cases

Around a third of the children (24/76; 31.6%) presented with highly invasive serotypes, out of which the most common were 14 (9/76; 11.8%), 3 (5/76; 6.6%), and 1 (5/76; 6.6%). Over half of the children (44/76; 57.9%) presented at least one serotype included in PCV13, and half of the children presented at least one serotype included in any of the two PCV10 (38/76 [50.0%] for Synflorix® and 37/76 [48.7%] for Pneumosil®) (Fig. 3).

## Discussion

We found a prevalence of PNC of 62.8%, which is comparable to that of other developing countries before the introduction of PCV [6, 17, 41]. Similar studies in India found a PNC prevalence ranging between 35 and 75% [41, 42]. We found no published data on PNC in children admitted with clinical pneumonia from other neighbouring countries. We used PCR to detect PNC, which is more sensitive than culture, and thereby which could lead to a higher prevalence as compared to pneumococcal carriage studies that used culture. However, the recent multicentric study conducted in nine settings over eight developing and emerging countries also used PCR and found an overall PNC prevalence of 68.1% among children admitted with suspected pneumonia, similar to our study [41]. As expected, prior administration of antibiotics in our study appeared to reduce pneumococcal carriage detection [17, 43].

The most prevalent serotypes were 7B/7C/40 (indistinguishable by laboratory technique) identified in 43.4% of the children, followed by 6A/B (15.8%), 14 (11.8%), 23F (7.9%), 3 and 1 (6.6% each). This serotype distribution is rather different from that described in similar studies. We found a much higher proportion of children with the serotype 7B/7C/40, while the proportion of children we identified with the serotypes 19F, 6A or 6B was considerably lower than in similar studies (proportion of serotypes in children sick with respiratory symptoms) [7, 17, 41, 42, 44] or among healthy children in community-based carriage studies [43, 45–48]. In the neighbouring context of India, 6A/B and 19F were also found to be the most prevalent serotypes in children admitted with clinical pneumonia, together with serotypes 14 and 23F. In a systematic review that included Indian studies looking at prevalence of serotype distribution among children with invasive pneumococcal disease prior to introduction of PCV, the most prevalent serotypes in decreasing order were 14, 1, 19F, 6B, 5, 6A, 9 V and 23F [49]. However, these were isolated from normally sterile sites such as blood, cerebrospinal fluid or pleural fluid. Data from other neighbouring countries are scarce, except for some data on pneumococcal serotype distribution among healthy children in Nepal [50, 51]. In our study, 19F and 6A/B were identified in 4/76 (5.3%) and 12/76 (15.8%) of the children, respectively. The proportion of the serotypes 14 and 23F, however, was similar to other studies. Serotype 1 has been identified as an important cause of highly invasive pneumococcal disease and is atypically found in carriage studies of healthy children [49, 52]. In our study, serotype 1 was the fifth most common serotype (together with serotype 3) identified, which is in line with findings of PNC in children sick with pneumonia.

PCV13, which was introduced in Bhutan after the end of the study, could potentially prevent the infection by at least one serotype in up to 57.9% of the children recruited in this study. Similar studies including two from India showed incongruences in these estimates, reporting PCV13 coverage ranging between 32 and 84% [7, 41, 42]. However, this is in line with the different serotype distribution we described above, as the most commonly identified serotypes (19F, 6A and 6B) in other studies are covered by PCV13, while the commonly identified serotypes 7B/7C/40 in our study are not.

Bacterial pneumonia secondary to viral respiratory infections such as influenza have been previously well characterized, and *S. pneumoniae* is the most common bacteria involved [53]. The nasopharynx is colonised by many bacteria and viruses and while the interaction between respiratory viruses and *S. pneumoniae* is likely to play a crucial role on the progression to the disease, this is a less well understood area [6, 7]. In the present study, respiratory viruses were detected in similar proportion of children with and without PNC. However, when looking at specific viral infections, children with PNC were less likely to be co-infected with rhinovirus (28.6% versus 47.5%; *p* = 0.046), and there was a trend of higher proportion of co-infection with RSV among colonized children (50.0% versus 32.5%; albeit non statistically significant, *p* = 0.075). Positive association of pneumococcal colonisation with influenza, RSV, adenovirus and rhinovirus have been found by previous studies, but findings have not been consistent [54–56]. Differences between studies might be explained by different pneumococcal serotype distribution as well as different circulation of viruses depending on seasonality and local epidemiology. One of the best understood and well documented synergistic viral-bacterial interaction is that of the influenza virus, identified as a risk factor for the acquisition, colonization and development of pneumonia due to *S. pneumoniae* [53, 55, 57–59]. Conversely in our study, presence of influenza virus was not related with PNC. This might be due to the small number of children with influenza and by the fact that this is a cross-sectional study with collection of NPW specimen at the time of admission, while bacterial superinfection is often sequential, occurring a few days after the viral episode.

This study has a number of limitations. These data and the statistically significant differences identified need to be interpreted with caution, due to the relatively small number of children enrolled. NPW samples were unavailable (not collected or insufficient for testing) for one third of children. However, children with and without NPW findings did not significantly differ in regard to baseline characteristics, evolution and outcomes (analysis not shown), except for severity of pneumonia during admission (99/121 [81.8%] in children with NPW findings versus 65/68 [95.6%] in children without; *p* = 0.007). Our findings relate to children admitted with clinical pneumonia and as such, they do not reflect PNC rate and pneumococcal serotype distribution in healthy children. This is a cross-sectional survey, where we collected NPW once (upon admission or as soon as possible after enrolment). Therefore, we did not pretend to determine whether viral infection precedes pneumococcal colonization or to evaluate their impact in the development of pneumonia. Longitudinal cohort studies would be required to address these questions.

## Conclusions

This study provides baseline information on the status of pneumococcal carriage among sick Bhutanese children just before the introduction of the pneumococcal vaccine, which is valuable to monitor its impact. Most common serotypes identified were 7B/7C/40, 6A/B, 14 and 23F, which differs from comparable studies and neighbouring countries. PCV13 has a potential coverage of at least one serotype presented by over half of the children, suggesting a role for the vaccine to reduce the burden associated with *S. pneumoniae*.

## Data Availability

The datasets used and/or analysed during this current study are available from the corresponding author on reasonable request.

## Abbreviations

CRP: C-reactive protein
CXR: Chest radiography
ESR: Erythrocyte sedimentation rate
HDU: High dependency unit
Hib: Haemophilus influenza type b
IBD: Invasive Bacterial Disease
IQR: Interquartile range
JDWNRH: Jigme Dorji Wangchuck National Referral Hospital
LMIC: Low- and middle-income countries
NA: Not applicable
NPW: Nasopharyngeal washing
PCV: Pneumococcal conjugated vaccine
PICU: Paediatric intensive care unit
PNC: Pneumococcal nasopharyngeal colonization
RIBhuC: Respiratory Infection in Bhutanese Children
RSV: Respiratory Syncytial Virus
RT-PCR: Real time polymerase chain reaction
SpO2: Peripheral capillary oxygen saturation
